# EFTUD2 maintains the survival of tumor cells and promotes hepatocellular carcinoma progression via the activation of STAT3

**DOI:** 10.1038/s41419-020-03040-5

**Published:** 2020-10-06

**Authors:** Mengxian Tu, Lu He, Yang You, Jinying Li, Nan Yao, Chen Qu, Wei Huang, Leibo Xu, Rongcheng Luo, Jian Hong

**Affiliations:** 1 Department of Pathophysiology, School of Medicine, Jinan University, 510630 Guangzhou, Guangdong China; 2 Guangdong Provincial Key Laboratory of Malignant Tumor Epigenetics and Gene Regulation and Department of Biliary-Pancreatic Surgery, Sun Yat-sen Memorial Hospital, Sun Yat-sen University, 510120 Guangzhou, Guangdong China; 3 Integrated Hospital of Traditional Chinese Medicine, Southern Medical University, 510315 Guangzhou, Guangdong China; 4 Department of Radiotherapy, Affiliated Cancer Hospital & Institute of Guangzhou Medical University, 510095 Guangzhou, Guangdong China; 5 Department of Gastroenterology, The First Affiliated Hospital of Jinan University, 510630 Guangzhou, Guangdong China

**Keywords:** Liver cancer, Cell death, Cell migration, Prognostic markers

## Abstract

Elongation factor Tu GTP binding domain containing 2 (EFTUD2), a spliceosomal GTPase, plays a pivotal role in multiple organ development and innate immune. It has been reported that EFTUD2 is a new host factor with activity against HCV infection. However, the role of EFTUD2 in solid tumors, including hepatocellular carcinoma (HCC), remains unexplored. In this study, we investigated the molecular function of EFTUD2 in HCC. Data from The Cancer Genome Atlas (TCGA) indicated an upregulation of EFTUD2 in HCC tissues compared to that in nontumor liver tissues. Immunohistochemical analysis performed on two independent HCC cohorts confirmed the upregulation of EFTUD2 in HCC tissues and further suggested that a high level of EFTUD2 expression predicted shorter overall and recurrence-free survival in HCC patients. Functional studies suggested that siRNA interference with EFTUD2 expression significantly suppressed cell viability, blocked cell cycle progression, facilitated tumor cell apoptosis, and inhibited metastasis, while the enhancement of EFTUD2 expression promoted the proliferation and migration of HCC cells both in vitro and in vivo. Surprisingly, we also found that the stable knockdown of EFTUD2 expression via lentivirus infection was lethal for HCC cells. This finding suggested that EFTUD2 was essential for maintaining the survival of HCC cells. Mechanistically, RNA sequencing and gene set enrichment analysis (GSEA) suggested that the gene sets of epithelial–mesenchymal transition (EMT) and the JAK/STAT3 pathway were enriched in EFTUD2-overexpressing cells. Further verification indicated that EFTUD2-overexpressing cells exhibited an EMT-like phenotype and had enhanced STAT3 activation, while the STAT3 inhibitor S3I-201 partially blocked these pro-malignant effects of EFTUD2 overexpression. In summary, we report EFTUD2 as a novel oncogene that helps to maintain the survival of HCC cells and promotes HCC progression through the activation of STAT3. The high level of expression of EFTUD2 in HCC tissues indicates shorter overall and recurrence-free survival in HCC patients.

## Introduction

Hepatocellular carcinoma (HCC) is an aggressive disease and one of the leading causes of cancer-related mortality worldwide^1,2^. In spite of significant technical improvements in curative treatments such as surgical resection and transplantation, the prognosis of patients with HCC remains poor. Recurrence, metastasis and limited drugs remain the major obstacles to recovery for HCC patients after surgical resection^3,4^. Thus, it is important to understand the molecular mechanisms underlying HCC progression for the development of earlier screening markers and therapeutic targets.

EFTUD2 is a highly conserved spliceosomal GTPase that plays a crucial role in diverse biological functions, including developmental defects^5,6^, spliceosome activation^7^, and immune responses^8,9^. Previous studies have identified a disease-causing role for EFTUD2 in polydysplasia, including mandibulofacial dysostoses^10^, dysplastic ears^11^, microcephaly, and intellectual disabilities^12,13^, and esophageal atresia^14,15^. Meanwhile, it has been reported that direct interaction between SNW1 and EFTUD2 is essential for cell survival in breast cancer. Deletion of EFTUD2 inhibits the association of endogenous proteins, leading to increased apoptosis in breast cancer cells^16^. Moreover, mutation in the *eftud2* gene has been shown to cause increasing neural precursor cell apoptosis and mitosis^17^. However, the expression and function of EFTUD2 in HCC is unknown.

In this study, we explored the clinical relevance and potential role of EFTUD2 in HCC. We found that EFTUD2 was significantly upregulated in HCC tissues and was necessary for the survival of HCC cells. Subsequent in vitro and in vivo studies suggested that EFTUD2 induced the epithelial–mesenchymal transition (EMT) of HCC cells via the activation of STAT3. Our findings suggest that EFTUD2 acts as an oncogene to help promote the survival and metastasis of HCC cells, which provides a further understanding of the underlying pathogenesis of HCC.

## Materials and methods

### Clinical samples and immunohistochemistry staining

We analyzed the features of HCC recurrence with a tissue microarray that included 90 patients (OUTDO Biotech, Shanghai, China), as well as a cohort including 126 patients who had undergone curative liver resection at the Affiliated Tumor Hospital of Guangzhou Medical University between September 2006 and June 2010. In addition, twenty normal hepatic tissues, obtained from patients who underwent resection due to the presence of benign hepatic lesions, were used as normal controls. Another 50 HCC specimens were collected from the First Affiliated Hospital of Jinan University. All experiments involving human tissues were approved by the research and ethics committee of the Affiliated Tumor Hospital of Guangzhou Medical University, informed consent was obtained from each patient.

Tissue sections were deparaffinized in xylene and rehydrated with ethanol, and blocking of endogenous peroxidase activity with 3% hydrogen peroxide was followed by microwaving in 0.01 M sodium citrate buffer for antigen retrieval, after which the slides were preincubated in 10% normal goat serum for 1 h, followed with incubation overnight at 4 °C with the following primary antibodies: EFTUD2 (1:200, Novus, Dallas, TX, USA,NB100-40849), Ki67 (1:200, ZSGB, Beijing, China, ZM0166), E-cadherin (1:200, Cell Signaling Technology, Beverly, MA, USA, 3195), vimentin (1:200, Cell Signaling Technology, 5714), and pSTAT3 (1:200, Cell Signaling Technology, 9145). Afterwards, the expression of the indicated proteins was detected by a horseradish peroxidase detection system according to the manufacturer’s instructions (DAKO, Glostrup, Denmark). The scores were independently rendered by two pathologists. Both the intensity and extent of immunostaining were taken into consideration, and the median IHC score (1.5) was chosen as the cut-off value for defining high and low expression.

### Cell culture and transfection

HCC cell lines Hep G2, Hep3B, and Huh7 were maintained in Dulbecco’s modified Eagle’s medium (Gibco, Gaithersburg, MD, USA) supplemented with 10% fetal bovine serum (FBS, Hyclone, Logan, UT) and 1% penicillin streptomycin (Gibco) in a humidified incubator at 37 °C and 5% CO_2_. The short interfering RNAs (siRNA) were synthesized by Ribobio (Guangzhou, China). Lipofectamine 3000 (Invitrogen, USA) was used to transfect the EFTUD2 and negative control siRNAs into the cells according to the manufacturer’s suggestions. To establish stably transfected cells, the cells were transfected with an EFTUD2 overexpression vector or shRNA using polybrene according to the manufacturer’s instructions and then selected in puromycin (2 μg/mL, GeneChem, Shanghai, China) for 1 week.

### RNA extraction and quantitative real-time PCR

Total RNA was extracted from the indicated cells by using the TRIzol reagent (Takara, Guangzhou, China); RNA was reverse transcribed to cDNA templates using random hexamers and a PrimeScript 1st Strand cDNA Synthesis Kit (Takara). The SYBR Green qPCR mix (Takara) was used for RT-qPCR. Assays were performed according to the manufacturer’s suggestions. The PCR primers were synthesized by Thermo Fisher (Guangzhou, China) and are shown in Supplementary Table 1. The results of the RT-qPCR were analyzed by 2−∆∆CT. Each experiment was performed independently in triplicate.

### Western blot analysis

Total proteins were extracted from the indicated cells with RIPA lysis buffer (Invitrogen, Carlsbad, CA, USA). The protein concentration was measured using the BCA protein assay kit (Pierce, Rockford, IL, USA). Western blotting was performed using a sodium dodecyl sulfate polyacrylamide gel electrophoresis (SDS-PAGE) electrophoresis system. The primary antibodies used for the western blots were as follows: rabbit polyclonal antibodies against EFTUD2 (Novus), vimentin (Cell Signaling Technology), GAPDH (Cell Signaling Technology, 5174), cleaved-caspase 3 (Cell Signaling Technology, 9661), cleaved-caspase 7 (Cell Signaling Technology, 8438), PARP (Cell Signaling Technology, 9532), MCL-1 (Cell Signaling Technology, 94296), and pSTAT3 (Cell Signaling Technology, 9145) and monoclonal antibodies against E-cadherin (BD Biosciences, San Jose, CA, USA, 564186), Twist1 (Abcam, Cambridge, UK, ab50887) and STAT3 (Cell Signaling Technology, 12640).

### Cell proliferation and colony formation assays

Over 3000 indicated cells were seeded in 96-well plates and incubated until they attached to the wells; after 2.5 h of incubation with 10 μL of Cell Counting Kit-8 (CCK8) reagents (Dojindo, Kumamoto, Japan) at the given time points, absorbance at 450 nm was measured. Three replicates were used for each experiment.

For colony-formation assays, the indicated cells were collected and seeded in 6-well plates at a density of 500 per well, followed by incubation at 37 °C in 5% CO_2_ for 14 days. The colonies were fixed with methanol and stained with 0.1% crystal violet for 15 min, after which they were counted.

### EdU incorporation assays

The indicated cells were seeded in 96-well plates at a density of 1 × 10^4^ per well; after 24 h, the cells were then incubated in 100 μL of medium with 5-Ethynyl-2′-deoxyuridine (EdU) for 2 h before fixing with 4% paraformaldehyde. The EdU was detected using the Cell-Light ™ EdU Apollo567 In Vitro Flow Cytometry Kit (Ribobio) according to the manufacturer’s protocol. Slides from the same experiment were imaged using the same settings. Three fields were chosen randomly to count the EdU-positive cells.

### Cell apoptosis

Briefly, the cells were harvested and washed in cold phosphate-buffered saline (PBS) twice, after which 100 µL 1× binding buffer was added to each sample, followed by incubation with 5 µL Annexin V-FITC and PI solution (Dojindo, Kumamoto, Japan) for 15 min at room temperature. The cell apoptosis rate was analyzed by flow cytometry after the addition of 300 µL 1× binding buffer.

To assess mitochondrial membrane potential, the indicated cells were seeded in 6-well plate; after 12 h, the cells were incubated with JC-1 working solution (Dojindo) for 45 min before washing in cold PBS twice, followed by the addition of imaging buffer solution. The cells were observed under a fluorescence microscope or analyzed by flow cytometry.

### Cell cycle analysis

The cell cycle was analyzed using the BD Cycletes Plus DNA Kit (BD Biosciences) according to the manufacturer’s instruction. Briefly, the cells were harvested and resuspended by adding 1 mL of solution while vertexing twice, after which the cells were stained with solution A, B, or C for 10 min, respectively. The stained cells were analyzed by flow cytometry at a fluorescence emission of 530 nm.

### Cell migration and invasive assays

Cell motility was assessed by cell migration and invasion assays using Transwell chambers with or without Matrigel (BD Biosciences). Cells (1 × 10^5^) suspended in 200 µL of serum-free medium were seeded in the upper chambers, and the lower chambers were filled with 500 µL medium containing 20% FBS. After incubation for 15–24 h (for migration) and 24–48 h (for invasion), the cells in the upper chambers were removed, and the cells that migrated or invaded were incubated in medium with one drop of Hoechst 33342 (Thermo Fisher Scientific, Waltham, MA) for 20 min, followed by counting under an inverted fluorescence microscope.

### Animals

To establish subcutaneous xenograft tumors, 4 × 10^6^ cells suspended in 200 µL of PBS were injected into the right flank of mice (total of 20 mice were randomly allocated to two group). After 6 days, the tumor volumes were measured and recorded every 3 days; the tumors were resected from the mice, weighed and photographed 4 weeks after injection. For the lung metastasis model, 6 × 10^6^ cells resuspended in 50 µL of PBS were injected into nude mice through the tail vein (total of 12 mice were randomly allocated to two group). All mice were killed 6 weeks later, and the lungs were resected for counting metastatic nodules. No blinding was applied in animal experiments. The 4–6 week-old male BALB/c athymic nude mice were purchased from the Guangdong Medical Lab Animal Center. All animals were maintained in specific pathogen-free conditions. All animal studies were conducted in accordance with the principles and procedures outlined in the Southern Medical University Guide for the Care and Use of Animals.

### RNA-seq analysis

Total RNA extracted from EFTUD2 overexpressed Hep G2 and control were submitted for RNA sequencing. The total RNA was extracted using the TRIzol reagent (Takara, Guangzhou, China), RNA concentration and quality were evaluated using Agilent 2100 Bioanalyzer system (Agilent Technologies, Santa Clara, CA). The mRNA was enriched with poly-A selection and the samples were sequenced on BGISEQ-500 platform (BGI Genomics, Shenzhen, China). The raw data were deposited in Gene Expression Omnibus (GEO) under accession number GSE154438.

### Statistical analysis

The data are presented as the mean ± standard deviations (SD) from three independent experiments. SPSS 22.0 and Prism 7.0 software were used to perform the statistical analysis. The Kaplan–Meier method was used to determine survival probability, and the differences were assessed by the log-rank test. The Student’s *t*-test was performed to assess the values between two groups with similar variance, and analysis of variance (ANOVA) was performed for analysis among multiple groups. Pearson Chi-squared test was used to evaluate the correlation between EFTUD2 and pSTAT3 expression, All data were meet the assumptions of the tests and the appropriate statistical methods was used. *P* values were considered statistically significant at *P* < 0.05.

## Results

### EFTUD2 is upregulated in HCC tissues

**Fig. 1:**
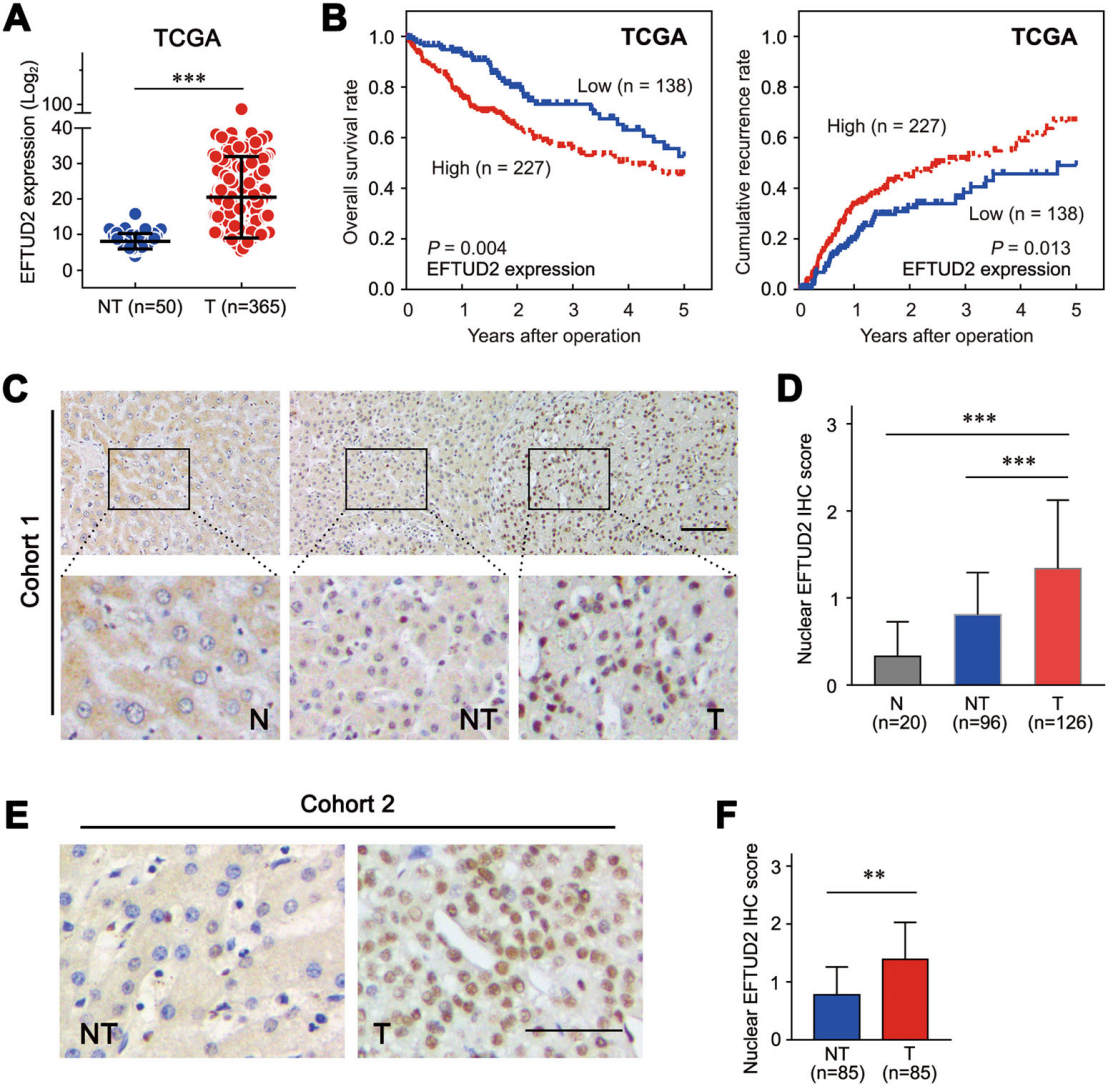
EFTUD2 is upregulated in HCC tissues. **a** The mRNA level of EFTUD2 expression among adjacent nontumor and HCC tissues (data from TCGA). **b** Kaplan–Meier curves for OS and TTR according to the EFTUD2 expression in HCC samples from the TCGA database. **c** Representative images of IHC staining for EFTUD2 in cohort 1, including normal hepatic (N, *n* = 20), adjacent nontumor (NT, *n* = 96) and HCC tissues (T, *n* = 126). Scale bars, 50 μm. **d** Nuclear EFTUD2 expression levels (IHC score) were compared among normal hepatic, adjacent nontumor and HCC tissues. **e** Representative images of IHC staining for EFTUD2 in cohort 2. Scale bars, 25 μm. **f** Nuclear EFTUD2 expression levels (IHC score) were compared among adjacent nontumor and HCC tissues. The data are presented as the mean ± SD. **P < 0.01, ***P < 0.001.

To determine the role of EFTUD2 in HCC, we first analyzed the mRNA expression of EFTUD2 in 365 primary HCC tissues and 50 nontumor tissues from the HCC dataset of the Cancer Genome Atlas (TCGA). We found that EFTUD2 was significantly upregulated in HCC tissues, compared to nontumor tissues (*P* < 0.001; Fig. 1a). The prognostic implication of EFTUD2 in HCC was explored next. Kaplan–Meier survival analysis revealed that patients with a high level of EFTUD2 expression exhibited a significantly poorer overall survival time and time to recurrence (Fig. 1b) compared with patients with a low level of EFTUD2 expression. We further explored the expression status of the EFTUD2 protein in HCC tissues. IHC analysis showed a high level nuclear-localized EFTUD2 expression in 42.6% (54/126) of the HCC tissues but in only 9.4% (9/96) of the nontumor tissues. We did not detect a significant nuclear-localized EFTUD2 expression in normal liver tissues (Fig. 1c, d and sFig.1). In addition, the IHC assay performed on another HCC cohort including 85 paired HCC and adjacent nontumor tissues further confirmed the upregulation of EFTUD2 in HCC tissues (Fig. 1e, f). These data collectively demonstrated that the upregulation of EFTUD2 is a frequent event in HCC and may serve as a promising factor for the prognosis of patients with HCC.

### High EFTUD2 expression in HCC tissues predicts a poor prognosis in HCC patients

**Fig. 2:**
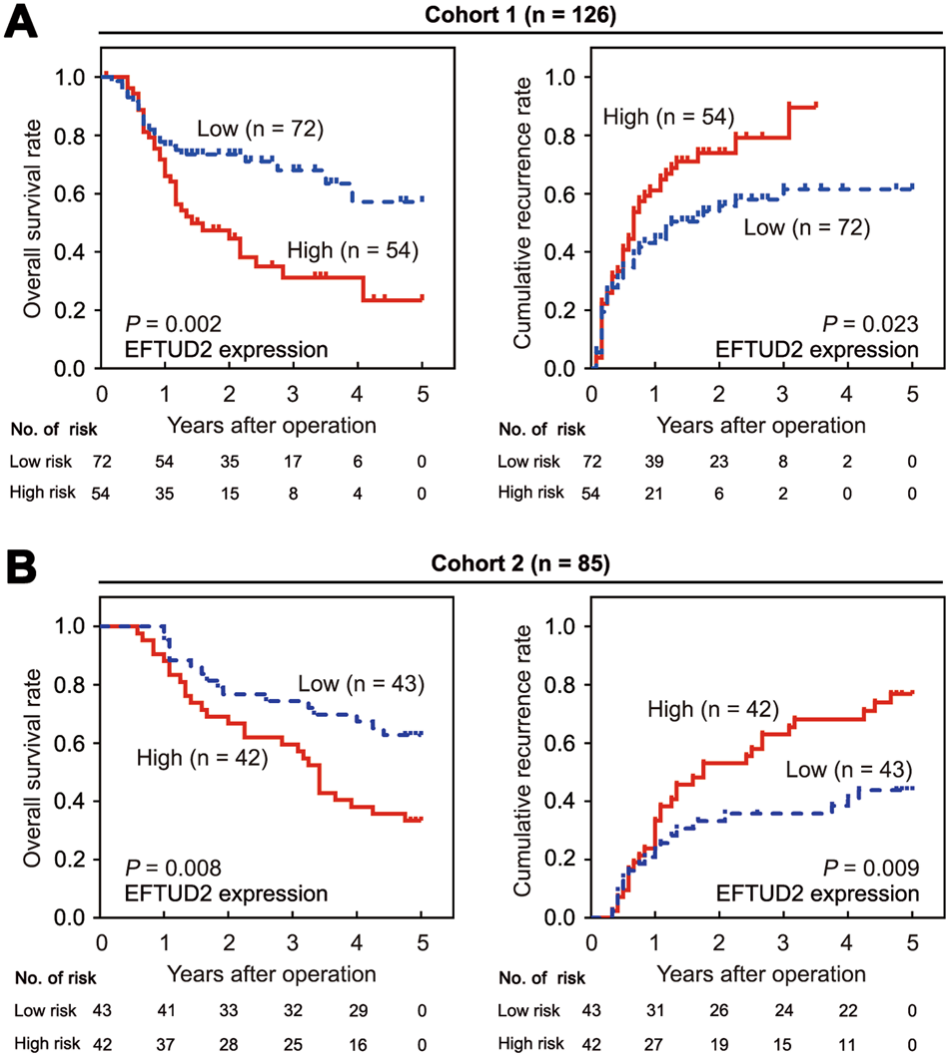
HCC EFTUD2 expression in HCC tissues predicts a poor prognosis in HCC patients. **a**, **b** Overall survival (OS) and cumulative recurrence rate of cohort 1 (*N* = 126) and cohort 2 (*N* = 85) HCC patients based on EFTUD2 expression in tumor tissues, respectively.

The prognostic value of EFTUD2 in the two HCC cohorts was next analyzed. The 5-year OS rate in patients with a high level of EFTUD2 expression was significantly lower than that in patients with a low EFTUD2 expression in the two independent cohorts (cohort 1: 23.3% versus 57.1%, cohort 2: 33.3% versus 62.8%; Fig. 2a, b). The 5-year recurrence rate in patients with a high level of EFTUD2 was also significantly higher than that in the patients with a low level of EFTUD2 expression (cohort 1: 89.6% versus 61.5%, cohort 2: 76.8% versus 43.8%; Fig. 2, b). The EFTUD2 high expression group had a significantly shorter OS and TTR than the EFTUD2 low expression group in two independent cohorts, which was consistent with the results of the TCGA data. Taken together, EFTUD2 is a powerful independent predictor of TTR and OS.

### EFTUD2 expression is necessary for the survival of HCC cells

**Fig. 3:**
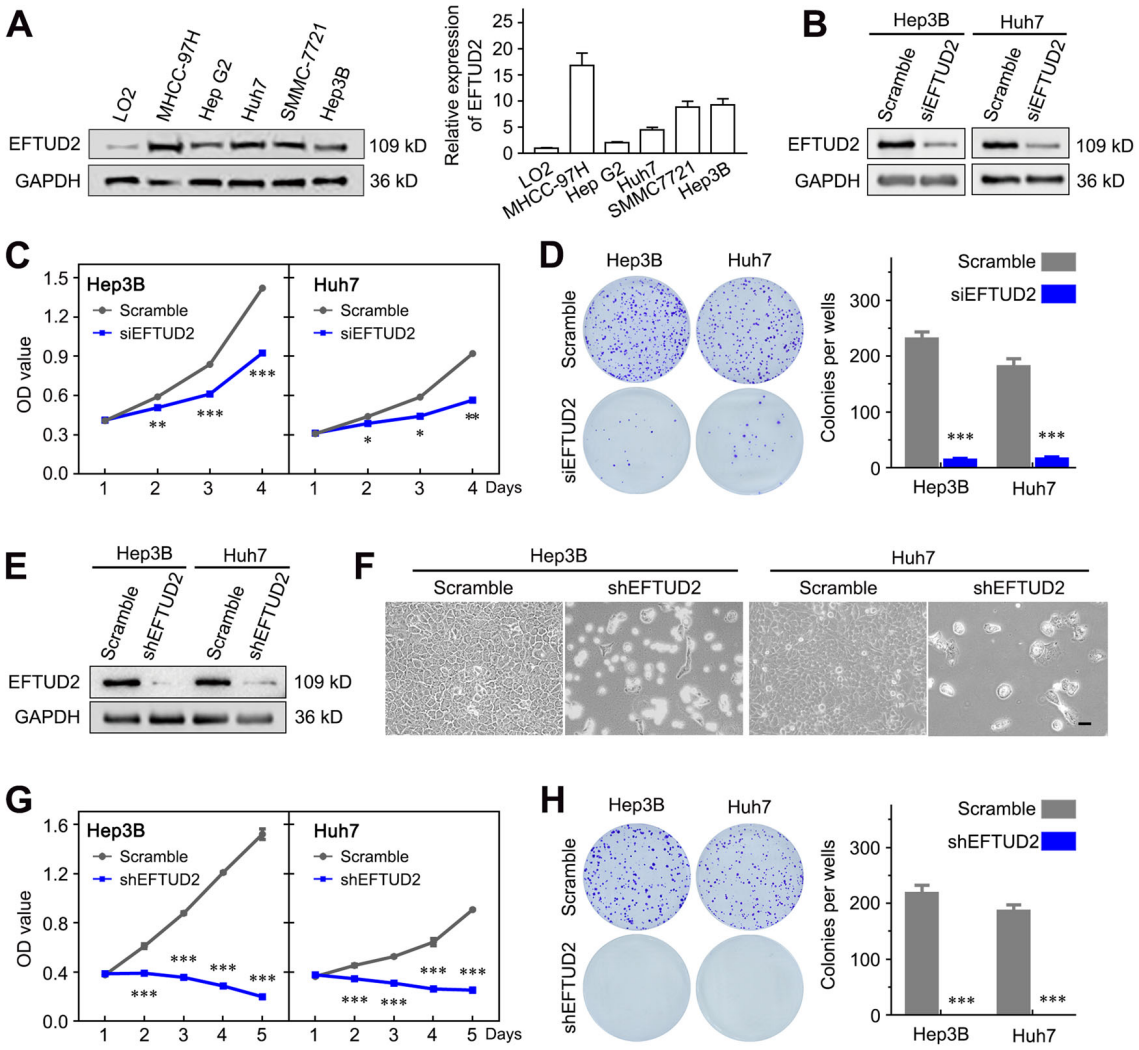
EFTUD2 expression is necessary for HCC cell survival. **a** The protein and mRNA levels of EFTUD2 expression in HCC cells detected by western blot and qPCR. **b** Western blot analysis of EFTUD2 expression in Hep3B and Huh7 cells infected with siRNA against EFTUD2 (siEFTUD2). **c. d** The effect of EFTUD2 on cellular viability was detected by CCK8 assays and colony formation assays, respectively. **e** Western blot analysis of EFTUD2 protein expression in Hep3B and Huh7 cells infected with lentiviruses expressing either scrambled shRNA or shRNA against EFTUD2 (shEFTUD2). **f** Representative images show the cell morphological changes after the stable knockdown of EFTUD2. Scale bars, 100 μm. **g**, **h** CCK8 assays and colony formation assays show the effect of EFTUD2 on cellular survival in Hep3B and Huh7 cells with a stable knockdown of EFTUD2. The data are presented as means ± SD. *P < 0.05; **P < 0.01; ***P < 0.001.

The functional role of EFTUD2 in HCC was next elucidated. RT-qPCR and western blot showed that the expression of EFTUD2 was significantly higher in a panel of HCC cell lines than that in the nontransformed hepatic cell line (LO2) (Fig. 3a). Hep3B and Huh7 were selected for the transient knockdown of EFTUD2 expression with siRNA targeting EFTUD2 (Fig. 3b). CCK8 and clone formation assays showed that EFTUD2 knockdown inhibits the cell growth and clone formation capacity of Hep3B and Huh7 cells (Fig. 3c, d). In addition, we stably knocked down EFTUD2 expression in Hep3B and Huh7 cells by infection with lentivirus vectors (Fig. 3e). Surprisingly, we found that the stable knockdown of EFTUD2 led to pronounced cell death in Hep3B and Huh7 cells (Fig. 3f). In addition, the EFTUD2 stable knockdown cells completely lost their proliferative and clone formation abilities (Fig. 3g, h). These results suggested that EFTUD2 is necessary for HCC cell survival.

### Depletion of EFTUD2 blocks cell cycle progression and promotes cell apoptosis

**Fig. 4:**
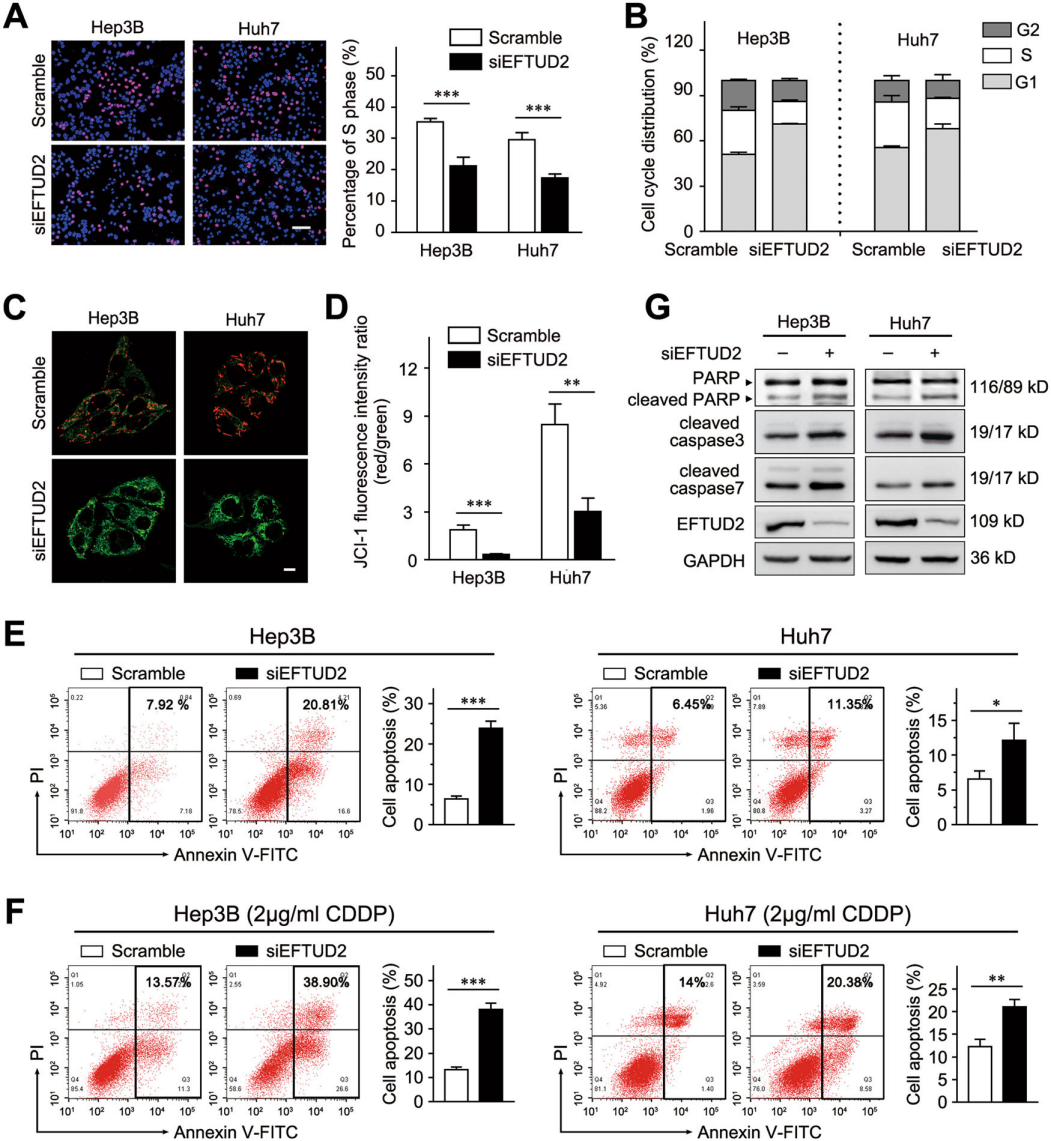
Depletion of EFTUD2 blocks cell cycle progression and promotes cell apoptosis. **a** EdU incorporation assays were used to identify the cells in S phase after EFTUD2 silencing in the indicated cells. Red: cell in S phase. Scale bars, 200 μm. **b** The cell cycle analyses were performed in Hep3B and Huh7 cells with EFTUD2 knockdown. **c**, **d** Fluorescence microscopy and flow cytometry were performed to captured early apoptotic cells by mitochondrial membrane potential changes in Hep3B and Huh7 cells with EFTUD2 knockdown. Red, JC-1 aggregates; green, JC-1 monomers. Scale bars, 10 μm. **e**, **f** EFTUD2-silenced Hep3B and Huh7 cells treated with or without CDDP (2 μg/mL; 48 h) were stained with a combination of Annexin V-FITC and PI and analyzed by FACS. Cells positive for FITC staining were counted as apoptotic cells. The bar graphs show the percentage of apoptotic cells. **g** Apoptosis-associated proteins of the indicated cells were measured by western blotting. The data are presented as the means ± SD. *P < 0.05; **P < 0.01; ***P < 0.001.

After observing the significant cell death after EFTUD2 depletion, we further investigated the potential functional role of EFTUD2 in cell cycle distribution and the apoptosis of HCC cells. Firstly, EdU incorporation and flow cytometry assays were performed to analyze the distribution of the cell cycle. We found that EdU-labeled cells were decreased in the EFTUD2-knockdown Hep3B and Huh7 cells, which indicated that EFTUD2 facilitated the S-phase transition (Fig. 4a). Consistent with the EdU assays, depletion of EFTUD2 in Hep3B and Huh7 cells decreased the percentages of cells in S phase (Fig. 4b). These results suggest that EFTUD2 knockdown obstructed the G1/S transition.

Next, we investigated the effect of EFTUD2 on HCC cell apoptosis by detecting mitochondrial membrane potential change. A stronger staining of the JC-1 monomer (green) and a weaker staining of J-aggregation (red) were observed in EFTUD2-knockdown Hep3B and Huh7 cells, indicating an early cell apoptosis (Fig. 4c). A similar result was detected by flow cytometry, which demonstrated that the fluorescence intensity ratio (red/green) was significantly decreased in EFTUD2-knockdown Hep3B and Huh7 cells (Fig. 4d). Moreover, FITC-based Annexin-V/PI double staining revealed that the percentages of apoptotic cells, including early and late, were significantly upregulated in the EFTUD2-knockdown Hep3B and Huh7 cells (Fig. 4e). In addition, the depletion of EFTUD2 also remarkably elevated CDDP-induced apoptosis in Hep3B and Huh7 cells (Fig. 4f). Furthermore, the expression levels of several key markers involved in apoptosis, including cleaved PARP, cleaved caspase 3, and cleaved caspase 7, were also significantly increased in EFTUD2-knockdown HCC cells (Fig. 4g). Taken together, these results indicate that EFTUD2 is a crucial molecule involved in cell cycle distribution and apoptosis in HCC cells.

### EFTUD2 promotes growth and invasion of HCC

**Fig. 5:**
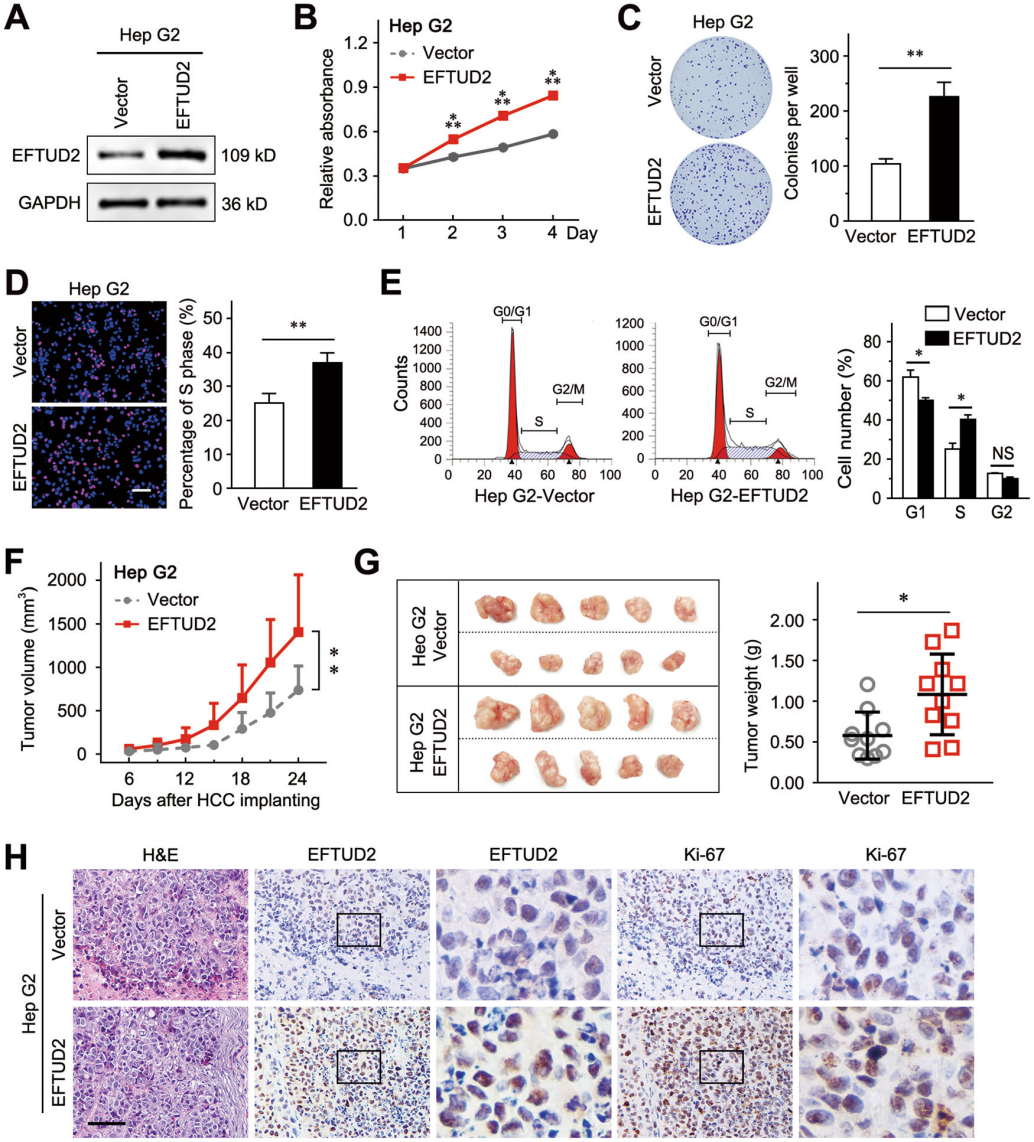
EFTUD2 promotes HCC growth. **a** Western blot analysis of EFTUD2 protein expression in Hep G2 cells infected with the EFTUD2 overexpression lentivirus or the vector lentivirus. **b**, **c** The effect of EFTUD2 overexpression on cell proliferation was detected by CCK8 assays and colony formation assays. **d** EdU incorporation assays were used to identify the cells in S phase. **e** The distribution of different cell cycle phases in the indicated cells. **f** Tumor volumes were measured and recorded every 3 days, and a growth curve was plotted. **g** Subcutaneous xenograft tumors of the indicated cells. The right panel shows the wet weights of the xenograft tumors. **h** Representative images of H&E and IHC staining of EFTUD2 and Ki67 in the xenograft tumors. Scale bars, 100 μm. The data are presented as the means ± SD. *P < 0.05; **P < 0.01; ***P < 0.001; NS no significance.

Because of the lethality of the stable EFTUD2 knockdown, we stably overexpressed EFTUD2 in Hep G2 cells to determine the regulatory function of EFTUD2 on HCC proliferation in vivo (Fig. 5a). The CCK8 and colony formation assays revealed that the ectopic expression of EFTUD2 promotes cell viability (Fig. 5b, c). Meanwhile, EdU incorporation and flow cytometry assays also indicated that EFTUD2 promotes cell cycle progression (Fig. 5d, e). Next, we established a subcutaneous transplant model in nude mice with EFTUD2-overexpressing Hep G2 cells. We found that the overexpression of EFTUD2 accelerated xenograft growth (Fig. 5f) and resulted in the apparent increase in the xenograft weights (Fig. 5g). In addition, IHC staining of EFTUD2 and the proliferating cell marker Ki-67 further confirmed the growth promoting effect of EFTUD2 (Fig. 5h).

**Fig. 6:**
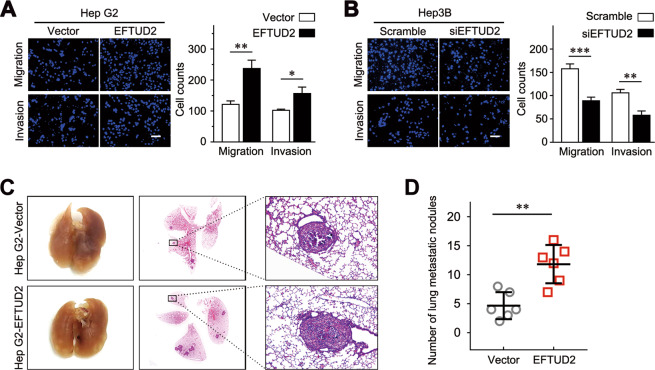
EFTUD2 promotes the migration and invasion of HCC cells. **a**, **b** Transwell assays were performed with Matrigel-coated or uncoated inserts to determine the migration and invasion capacities of the indicated cells. Scale bars, 200 μm. **c** Representative images of lung metastatic tumors obtained from an in vivo lung metastasis model established via the tail vein injection of tumor cells. **d** Lung metastasis nodules were counted and plotted (*N* = 6). The data are presented as the means ± SD. *P < 0.05; **P < 0.01.

Moreover, we also assessed whether EFTUD2 regulates the invasion and metastasis of HCC. Compared with the control cells, the ectopic expression of EFTUD2 significantly promoted cell migration and invasion in vitro (Fig. 6a). Conversely, the opposite results were observed when EFTUD2 was depleted in Hep3B cells (Fig. 6b). Moreover, data from an in vivo lung metastasis model (established via tail vein injection of cancer cells) suggested that the overexpression of EFTUD2 led to increased lung metastatic nodules (Fig. 6c, d). Taken together, these results indicated that EFTUD2 possesses oncogenic activities in HCC.

### EFTUD2 promotes EMT via activation of the STAT3 pathway in HCC cells

**Fig. 7:**
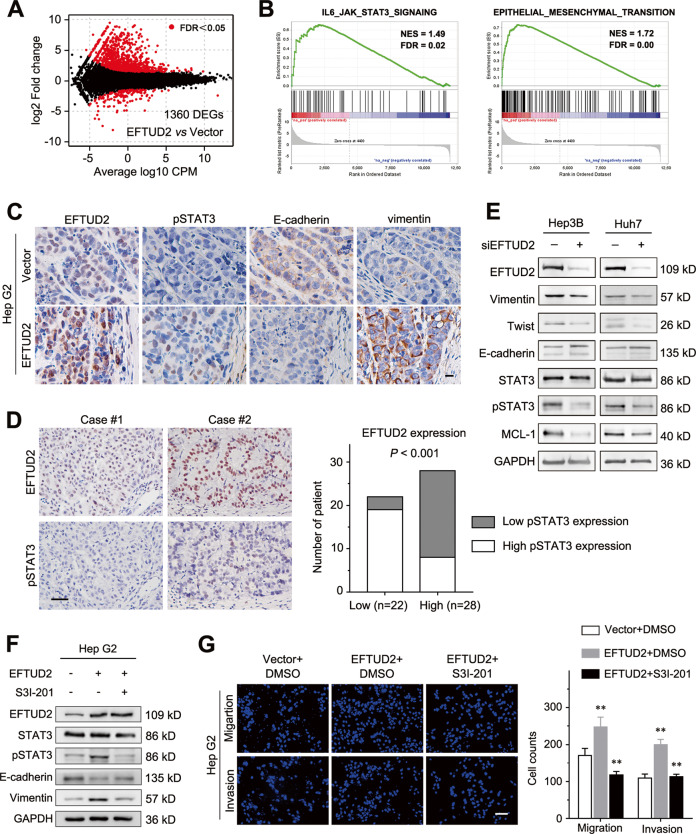
EFTUD2 promotes EMT via activation of the STAT3 pathway in HCC cells. **a** An M–A plot showed the genes differentially expressed between EFTUD2-overexpressing and control Hep G2 cells (*N* = 3), with the differentially regulated genes highlighted in red. **b** Gene sets of the IL6_JAK_STAT3 signaling pathway and the EMT were enriched in EFTUD2-overexpressing Hep G2 cells. **c** Serial sections of the xenograft tumors were subjected to IHC staining of EFTUD2, pSTAT3, E-cadherin and vimentin. Scale bars, 100 μm. **d** Serial sections of human HCC tissue samples were subjected to IHC staining with antibodies against EFTUD2 and pSTAT3. Representative IHC images were showed in the left panel, and the right panel showed the correlation between EFTUD2 and pSTAT3 expression, in 50 human HCC tissue samples. Scale bars, 100 μm. **e** The expression of EFTUD2, vimentin, Twist1, E-cadherin, STAT3, pSTAT3, MCL-1, and GAPDH was detected by western blotting in the indicated cells. **f** Western blot analysis showing that the increases in pSTAT3 and vimentin expression were compromised and that the decrease in E-cadherin was reversed by SI3-201, a STAT3 inhibitor, in EFTUD2-overexpressing Hep G2 cells. **g** The Hep G2 cells with EFTUD2 overexpression were treated with 100 μM SI3-201 and subjected to migration and invasion assays. Scale bars, 200 μm. The data are presented as the mean ± SD from triplicate independent experiments, **P < 0.01.

To explore the mechanism by which EFTUD2 regulated HCC proliferation and metastasis, we performed RNA-Seq on Hep G2 cells with a stable EFTUD2 overexpression. We found that the overexpression of EFTUD2 led to the significant differential expression of 1360 genes (Fig. 7a). We then performed a Gene Set Enrichment Analysis (GSEA) to identify pathway enrichment and determine the most significant molecular pathways regulated by EFTUD2. This analysis showed a significant enrichment of genes involved in the EMT and the JAK/STAT3 pathway, which play an essential role in the development of HCC by controlling cell proliferation, apoptosis, and motility (Fig. 7b). Among the differentially expressed genes involved in EMT, the changes were validated by qPCR (Suppl. Fig. 2). Meanwhile, data from TCGA showed that the expression levels of EFTUD2 positively correlated with MCL-1, an anti-apoptosis protein that is regulated by the STAT3 pathway^18,19^, as well as the EMT-related genes Twist1 and vimentin (Suppl. Fig. 3). Consistent with the RNA-Seq and GSEA results, the IHC assays performed on the serial sections of subcutaneous xenografts also showed that the EFTUD2 expression positively correlated with the expression of activated STAT3 (phosphorylated STAT3, pSTAT3) and the mesenchymal marker vimentin, but negatively correlated with the epithelial marker E-cadherin (Fig. 7c). Moreover, we analyzed the expression of EFTUD2 and pSTAT3 in serial sections of 50 human HCC samples and found that EFTUD2 expression significantly correlated with pSTAT3 expression in HCC specimens (Fig. 7d). In addition, we also demonstrated that knockdown of EFTUD2 remarkably inactivated STAT3, downregulated MCL-1, and reversed EMT (Fig. 7e and Suppl. Fig. 4). Emerging evidence has shown that STAT3 is a key oncogenic factor involved in the activation of several pathways, including cell proliferation and invasion, as well as cancer cell survival^20–22^. Moreover, the activation of STAT3 also promotes EMT of HCC cells^23^. These observations collectively indicated that EFTUD2 promotes cell survival, metastasis, and EMT primarily through STAT3 regulation.

To further investigate whether EFTUD2 mediated EMT through the activation of STAT3, EFTUD2-overexpressing Hep G2 cells were treated with an inhibitor of STAT3 (S3I-201). The results showed that the expression level of E-cadherin was restored, and that the expression level of vimentin was compromised by the STAT3 inhibitor in EFTUD2-overexpressing Hep G2 cells (Fig. 7f). In addition, the transwell assays showed that treatment with the STAT3 inhibitor also reversed the metastasis and invasion enhancement induced by EFTUD2 overexpression (Fig. 7g). Taken together, these results suggest that EFTUD2 promotes STAT3 activation, thus inducing EMT and promoting the metastasis of HCC cells.

## Discussion

Previously, there was little known about the molecular function of EFTUD2 in HCC development. In this study, we identified the exact role and related mechanism of EFTUD2 in HCC. We observed that nuclear EFTUD2 was upregulated in HCC tissues compared to adjacent nontumor and normal liver tissues. In addition, we found that EFTUD2 is an independent prognostic factor for HCC patients. A high level of EFTUD2 expression predicted a shorter overall and recurrence-free survival time in HCC patients.

Interestingly, we found that EFTUD2 likely plays a role in maintaining the survival of HCC cells. Our results indicated that a stable EFTUD2 knockdown with a lentivirus vector was lethal for two different HCC cell lines, while a transient knockdown of EFTUD2 with siRNA inhibited cell viability but was not lethal. The flow cytometry and mitochondrial membrane potential assay results confirmed that the depletion of EFTUD2 expression blocks cell cycle progression and promotes cell apoptosis. Meanwhile, apoptosis-related proteins, cleaved PARP, cleaved caspase 3, and cleaved caspase 7, were increased. Our data indicated that EFTUD2 knockdown decreased the cell number at S phase of cell cycle, while EFTUD2 overexpression increased the cells at S phase. These results suggested that EFTUD2 takes part in DNA replication of the cell cycle. We speculate that EFTUD2 knockdown and the serious DNA replication arrest leads to cell apoptosis. Further study to investigate the molecular mechanism of possible link between cell apoptosis, and S phase of cell cycle regulated by EFTUD2 in HCC is warranted. Moreover, our data suggested that the ectopic expression of EFTUD2 promotes cell proliferation and migration in vivo and in vitro.

Next, we explored the exact EFTUD2-associated regulatory mechanism in HCC cells. RNA-Seq was performed on Hep G2 cells overexpressing EFTUD2, and GSEA analysis showed that the JAK/STAT3 pathway and the EMT were closely related with EFTUD2. Our further studies confirmed that the phosphorylation of STAT3 was decreased, and MCL-1, a downstream gene regulated by the STAT3 pathway, was significantly decreased in the EFTUD2 knockdown cells. STAT3 has been reported to maintain the survival of cancer cells^24^. We found that EFTUD2-mediated HCC survival through STAT3. STAT3 activation induces the production of interferon, growth factors and several cytokines through a JAK-dependent manner^25^. We found that EFTUD2 promoted the phosphorylation of STAT3, but did not affect total STAT3 protein expression. Moreover, our RNA-sequencing data indicated that overexpression of EFTUD2 increased IL-6 expression. IL-6 is a well reported cytokine that activates STAT3 signaling pathway^26^. It has been reported that the depletion of IL6 expression blocks the activation of STAT3 pathway, and thus rescues the oncogenic effect of STAT3 signaling pathway2^22,27^. We therefore suggested that EFTUD2 promotes IL-6 production and STAT3 activation to maintain the survival of HCC cells. Depletion of IL6 expression would reduce the oncogenic effects of the EFTUD2. However, more detailed studies are needed to verify this effect. The EFTUD2 serves as a spliceosomal GTPase, which helps process mRNA. Although we observed that overexpression of EFTUD2 promoted IL-6 expression (Suppl. Fig. 2), there was no evidence to support that EFTUD2 overexpression affected IL-6 mRNA splicing. We speculated that EFTUD2 may up-regulate IL-6 expression via an indirect way. However, more studies about the detailed molecular mechanism are needed.

In addition, we found that EFTUD2 was also closely related with EMT, which indicates that the weakened cell invasion and metastasis abilities were not caused by cell apoptosis. The EMT, in which epithelial cancer cells lose their polarity and become motile mesenchymal cells, has been implicated in carcinoma invasion/metastasis^28,29^. The characteristic upregulation of E-cadherin, a key step of EMT, was also observed in the EFTUD2 knockdown cell lines. Growing evidence has indicated that several cancer-associated signaling pathways are involved in the regulation of EMT, including the JAK/STAT3 pathway^30,31^. Therefore, a STAT3 inhibitor was used to clarify the interaction between STAT3 and EMT. Transwell assays showed that enhanced EFTUD2 expression led to elevated metastatic potential but was compromised by the inhibitor. Meanwhile, the decreased level of E-cadherin was rescued by the inhibitor, while the increased level of vimentin was compromised.

Our study identified that a high level of EFTUD2 expression is a predictive biomarker for a poor prognosis in HCC. We also found that EFTUD2 sustains survival and promotes the EMT of HCC cells via the activation of STAT3. Our finding provides a new direction to our understanding of the survival and prognosis mechanism of HCC.

## Supplementary information

Supplementary Table 1

Supplemental Figures Legends

Supplementary Figure 1

Supplementary Figure 2

Supplementary Figure 3

Supplementary Figure 4
