# Supplementary material for: EFTUD2 maintains the survival of tumor cells and promotes hepatocellular carcinoma progression via the activation of STAT3
Source: Cell Death Dis. 2020 Oct 6;11(10):830. doi: 10.1038/s41419-020-03040-5 (PMC7538941; doi:10.1038/s41419-020-03040-5)
Supplement: Supplementary file 1 — Supplementary Table 1 [file 41419_2020_3040_MOESM1_ESM.docx]

**Supplementary Table 1**. Real-Time Polymerase Chain Reaction Primers

| Gene | Species | Primer sequence (5′ to 3′) |
| --- | --- | --- |
| *EFTUD2* | *Homo sapiens* | Forward: CAATATCATGGACACTCCAGGAC |
|  |  | Reverse: CGGTCAATCTTGTTGATGCACA |
| *GAPDH* | *Homo sapiens* | Forward: ATGACCCCTTCATTGACC |
|  |  | Reverse: GAAGATGGTGATGGGATTTC |
| *E-cadherin* | *Homo sapiens* | Forward: TCGACACCCGATTCAAAGTGG |
|  |  | Reverse: TTCCAGAAACGGAGGCCTGAT |
| *Vimentin* | *Homo sapiens* | Forward: TGGCCGACGCCATCAACACC |
|  |  | Reverse: CACCTCGACGCGGGCTTTGT |
| *Twist* | *Homo sapiens* | Forward: GTCCGCAGTCTTACGAGGAG |
|  |  | Reverse: GCTTGAGGGTCTGAATCTTGCT |
| *MCL-1* | *Homo sapiens* | Forward: CGACGGCGTAACAAACT |
|  |  | Reverse: GGAAGAACTCCACAAACCC |
| *PTGS2* | *Homo sapiens* | Forward: CAAACCTACGCCAAAATCCA |
|  |  | Reverse: GAAATGAATGAGCCTACAGA |
| *LUM* | *Homo sapiens* | Forward: TTGCGTTTGGATGGCAATCG |
|  |  | Reverse: GAGTGACTTCGTTAGCAACACG |
| *MMP1* | *Homo sapiens* | Forward: CTCTGGAGTAATGTCACACCTCT |
|  |  | Reverse: TGTTGGTCCACCTTTCATCTTC |
| *SPP1* | *Homo sapiens* | Forward: TGCCAGCAACCGAAGTTTTC |
|  |  | Reverse: TGTCAGGTCTGCGAAACTTC |
| *TGM2* | *Homo sapiens* | Forward: TGCCCTTTGGAAAGCCATTG |
|  |  | Reverse: TTTTTGCCTGCTCCAAGGAG |
| *TPM2* | *Homo sapiens* | Forward: AGAGGTCTGTGGCAAAGTTG |
|  |  | Reverse: AGGTTGTTGAGTTCCAGCAG |
| *IGFBP3* | *Homo sapiens* | Forward: GGGGTGTACACATTCCCAAC |
|  |  | Reverse: AGGCTGCCCATACTTATCCA |
| *IL-6* | *Homo sapiens* | Forward: ACTCACCTCTTCAGAACGAATTG |
|  |  | Reverse: CCATCTTTGGAAGGTTCAGGTTG |
