# Supplementary material for: EFTUD2 maintains the survival of tumor cells and promotes hepatocellular carcinoma progression via the activation of STAT3
Source: Cell Death Dis. 2020 Oct 6;11(10):830. doi: 10.1038/s41419-020-03040-5 (PMC7538941; doi:10.1038/s41419-020-03040-5)
Supplement: Supplementary file 2 — Supplemental Figures Legends [file 41419_2020_3040_MOESM2_ESM.docx]

**Supplementary Figures Legends:**

**Supplementary Figure 1. Nuclear EFTUD2 expression is upregulated in tumor tissues.**

The intensity of nuclear EFTUD2 expression is shown. Scale bars, 100 μm.

**Supplementary Figure 2. Part of the most significant upregulated genes identified by RNA-sequencing in EFTUD2 overexpressed Hep G2 cells are validated by qPCR.**

The data are presented as means ± SD. ***, *P* < 0.001.

**Supplementary Figure 3. EFTUD2 expression is positively correlated with MCL-1, TWIST1 and Vimentin in HCC samples (data from TCGA).**

**Supplementary Figure 4. EFTUD2 suppresses epithelial-mesenchymal transition (EMT) of HCC cells.**

The mRNA expression of E-cadherin, Vimentin, TWIST1 and MCL-1 of the indicated cells. The data are presented as means ± SD. *, *P* < 0.05; **, *P* < 0.01; ***, *P* < 0.001.
