# Supplementary figures and images for: EFTUD2 maintains the survival of tumor cells and promotes hepatocellular carcinoma progression via the activation of STAT3
Source: Cell Death Dis. 2020 Oct 6;11(10):830. doi: 10.1038/s41419-020-03040-5 (PMC7538941; doi:10.1038/s41419-020-03040-5)

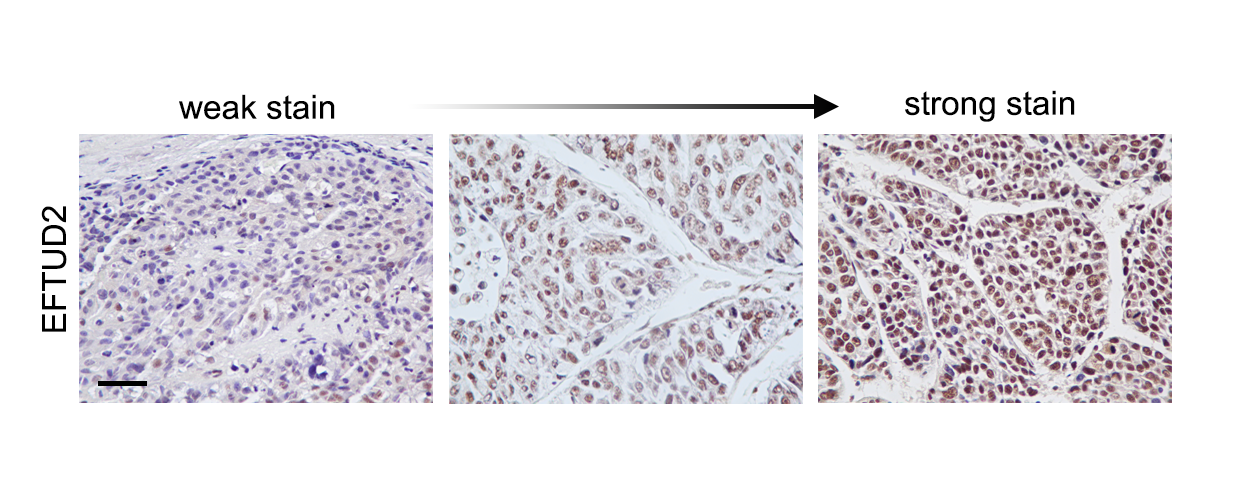

Supplement: Supplementary file 3 — Supplementary Figure 1 [file 41419_2020_3040_MOESM3_ESM.tif]

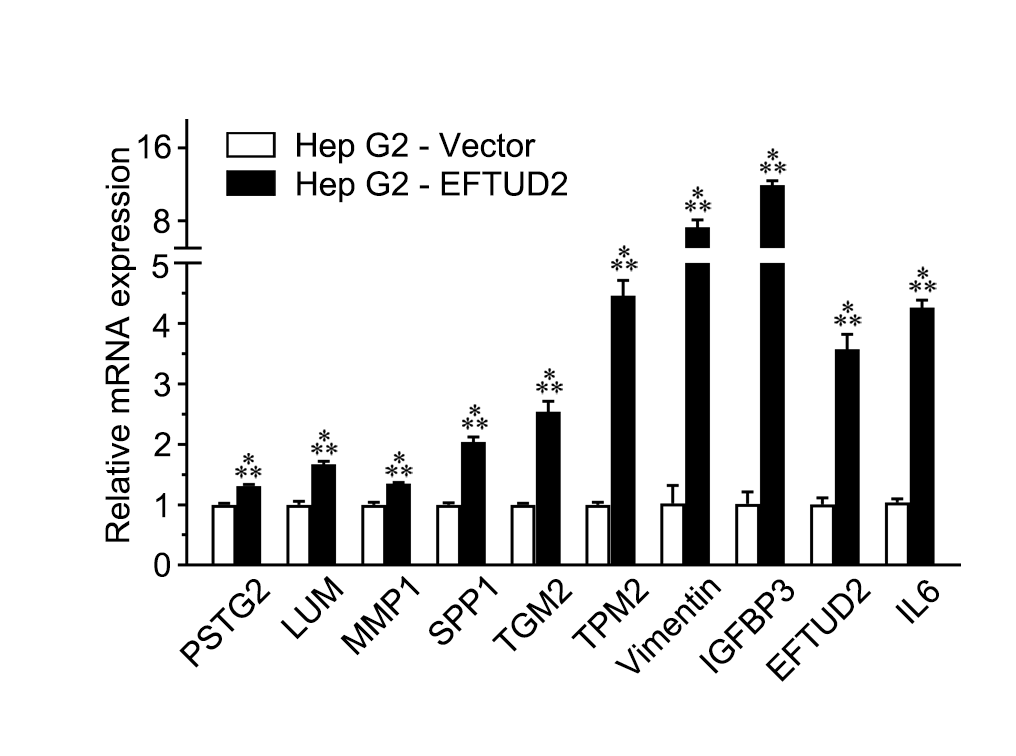

Supplement: Supplementary file 4 — Supplementary Figure 2 [file 41419_2020_3040_MOESM4_ESM.tif]

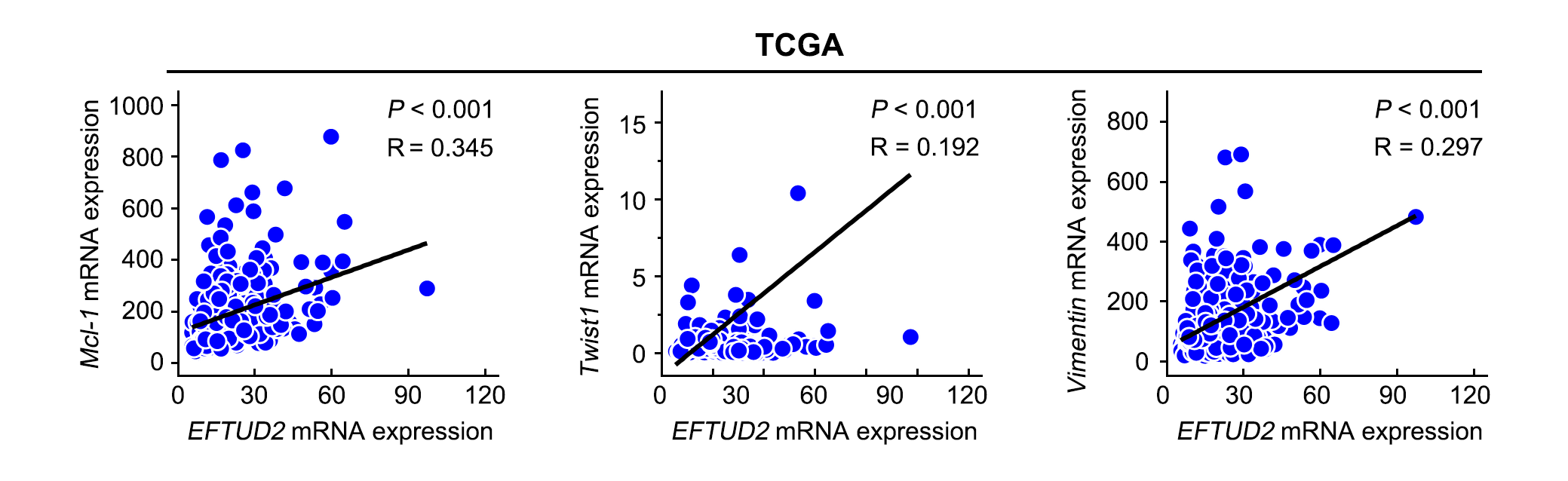

Supplement: Supplementary file 5 — Supplementary Figure 3 [file 41419_2020_3040_MOESM5_ESM.tif]

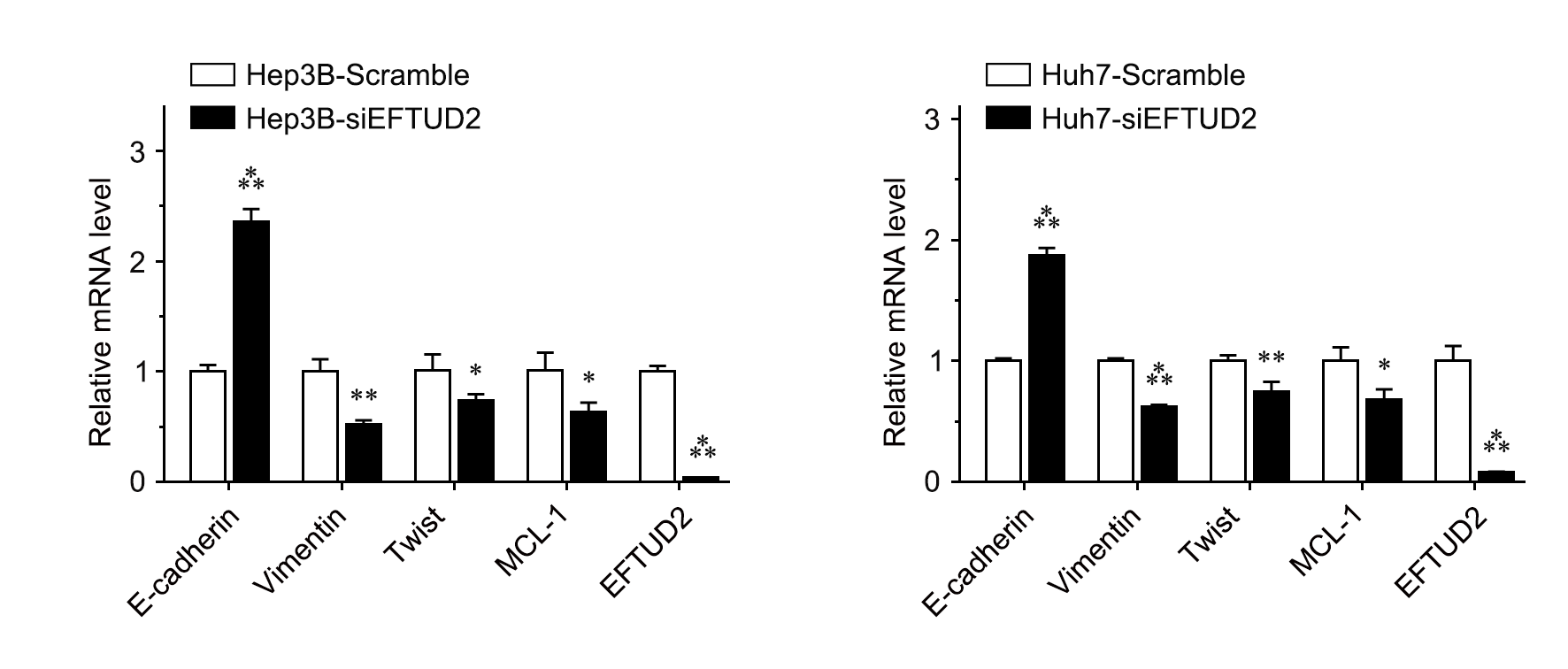

Supplement: Supplementary file 6 — Supplementary Figure 4 [file 41419_2020_3040_MOESM6_ESM.tif]
